# Benchmarking Metagenomic Classifiers on Simulated Ancient and Modern Metagenomic Data

**DOI:** 10.3390/microorganisms11102478

**Published:** 2023-10-02

**Authors:** Vaidehi Pusadkar, Rajeev K. Azad

**Affiliations:** 1 Department of Biological Sciences, University of North Texas, Denton, TX 76203, USA; vaidehipusadkar@my.unt.edu; 2 BioDiscovery Institute, University of North Texas, Denton, TX 76203, USA; 3 Department of Mathematics, University of North Texas, Denton, TX 76203, USA

**Keywords:** microorganisms, microbial DNA, ancient metagenomics, taxonomic profiling, benchmarking

## Abstract

Taxonomic profiling of ancient metagenomic samples is challenging due to the accumulation of specific damage patterns on DNA over time. Although a number of methods for metagenome profiling have been developed, most of them have been assessed on modern metagenomes or simulated metagenomes mimicking modern metagenomes. Further, a comparative assessment of metagenome profilers on simulated metagenomes representing a spectrum of degradation depth, from the extremity of ancient (most degraded) to current or modern (not degraded) metagenomes, has not yet been performed. To understand the strengths and weaknesses of different metagenome profilers, we performed their comprehensive evaluation on simulated metagenomes representing human dental calculus microbiome, with the level of DNA damage successively raised to mimic modern to ancient metagenomes. All classes of profilers, namely, DNA-to-DNA, DNA-to-protein, and DNA-to-marker comparison-based profilers were evaluated on metagenomes with varying levels of damage simulating deamination, fragmentation, and contamination. Our results revealed that, compared to deamination and fragmentation, human and environmental contamination of ancient DNA (with modern DNA) has the most pronounced effect on the performance of each profiler. Further, the DNA-to-DNA (e.g., Kraken2, Bracken) and DNA-to-marker (e.g., MetaPhlAn4) based profiling approaches showed complementary strengths, which can be leveraged to elevate the state-of-the-art of ancient metagenome profiling.

## 1. Introduction

The evolutionary history of genetic information of all extant microorganisms goes back up to billions of years in time. Advances in metagenome sequencing have enabled the reconstruction of evolutionary trajectories to understand how microbial communities have evolved through time. Such studies have highlighted the emergence and evolution of microbes involved in infectious diseases [1,2], ancient pandemics [3], and migrations that happened in the past. Ancient metagenomics refers to the analysis of complex genomic content which is retrieved from degraded biological material of ancient or extinct microorganisms. Research in this field is commonly focused on adding a new perspective to our understanding of the past and providing exciting sources of information for modern metagenomics studies. With the decrease in sequencing cost and increase in coverage depth, thanks to the next-generation high throughput sequencing technologies, reliable estimation of the relative abundance of organisms (or taxa) dwelling in an environment is now possible [4]. Popular methods for profiling microbial communities use sequence data obtained using either 16S rRNA amplicon sequencing or whole shotgun metagenome sequencing. The former targets the 16S rRNA gene for taxonomic profiling, while the latter targets the entire complement of genomic information in a community to profile at lower taxonomic levels including strain-level classification, as well as specifies genetic functions represented in a community [5,6,7]. With the decline in sequencing cost, the latter is becoming the more favored approach for profiling metagenomic data.

Simultaneously, advances in sequencing have contributed to reconstructing genomes from ancient DNA (aDNA) samples obtained from various sources [8,9,10]. This has spawned a plethora of opportunities in the field of metagenomics, specifically, the study of ancient microbiomes, ancient pathogens, and sedimentary DNA, and further, this is being leveraged to advance modern metagenomics [11,12]. This is spearheaded by Standards, Precautions, and Advances in Ancient Metagenomics (SPAAM—www.spaam-community.org (accessed on 15 September 2023), a community of collaborating researchers who have built a metadata repository containing ancient metagenomic data collected from various sources or sites [13].

Metagenomic profiling of ancient microbial DNA samples is, however, challenging due to the damages in aDNA collected over the years [13]. These damages include C/G-to-T/A misincorporations caused by deamination in the DNA fragments [14,15]. Furthermore, remnants of aDNA are often very short fragments, as short as 50 bp, which makes their profiling difficult [16,17]. In addition, contamination of aDNA with modern DNA substantially depletes endogenous metagenomic DNA recovered from a source [18]. These damages or contaminations can inflict major biases on population genetic and phylogenetic analyses. Laboratory-based protocols have been used to reduce the effects of deamination; this entails ancient DNA screening that requires producing only a single uracil–DNA–glycosylase (UDG) treated library per sample [19]. However, these protocols are not efficient in removing contaminants [19].

Metagenomic profilers designed for identifying and profiling organisms or taxa represented in metagenomic samples have been consistently improvised to interrogate complex metagenomic samples at different taxonomic resolutions and quantify the relative abundance of taxa (from phyla to strains) [20]. Underlying these profilers are distinct algorithms that aim to decipher taxa at higher resolution and with high accuracy and low computational requirements. One such algorithm is sequence alignment, in which the sequences are aligned against the reference database, and based on the successful match, the sequences are classified [21]. Other algorithms rely on the exact matching of *k*-mers (short substrings of length *k*) from metagenome reads to *k*-mers in sequences in the microbial database [22]. Tools using variants of these algorithms exist, e.g., DNA–DNA comparison methods, where similarity of metagenomic reads with DNA sequences in the microbial DNA database is assessed [22,23,24,25,26,27]; DNA–protein comparison methods, where protein products (translated in all six reading frames) of metagenomic reads are compared to protein sequences in the database [28,29,30]; and DNA–marker method, where only a subset of specific gene sequences (known as “markers”) from the reference database are used for alignment with the metagenomic reads and taxa are inferred based on the discriminatory power of specific gene (marker) families that they are uniquely associated [31,32]. Although these profilers have extensively been benchmarked on modern metagenomic samples (both real and simulated), their efficacy in classifying reads from ancient metagenomic samples has not yet been comprehensively evaluated.

MetaBIT [33] and HOPS [34] are among a few pipelines developed for ancient metagenomic profiling. MetaBIT employs the modern metagenomic profiler MetaPhlAn2 in its pipeline and has been tested on modern and ancient metagenomes; however, its performance relative to other frequently used metagenomic profilers has not been assessed. HOPS runs on MALT [35], which provides the options of polymerase chain reaction (PCR) duplicate removal and deamination pattern tolerance at the ends of reads. However, MALT requires high computational memory, which can go over 1 TB of RAM on even a modest size genome database to build and medium-size FASTQ files to input [36]. Therefore, it is often only used in research clusters capable of supporting and maintaining such large databases and allowing usage of large memory systems. This restricts its use by researchers who lack such resources and this, in effect, limits its broad use and thus its contributions to ancient microbiome profiling.

Researchers investigating ancient metagenomic samples have relied on modern metagenomic profilers for the analysis of these samples [17,37,38]. However, the efficiency of these tools in profiling ancient samples is largely unknown because of the lack of studies on benchmarking these tools on ancient metagenomes. Most benchmarking studies have assessed these algorithms on profiling modern metagenomic samples [20,39,40,41], and none of these studies have attempted to benchmark the algorithms on ancient metagenomes. It is, therefore, imperative to benchmark the effectiveness of modern metagenomic profilers on degraded ancient DNA samples. A handful of studies on benchmarking metagenomic profilers on ancient metagenomic DNA samples have appeared in recent years [42,43,44,45]. One of the first such studies to perform an assessment tested five metagenomic profilers, QIIME/UCLUST, MetaPhlAn2, MIDAS, CLARK-S, and MALT, on in silico datasets mimicking a dental plaque microbiome with or without aDNA damage [44]. A follow-up study assessed the nucleotide (translated product) versus amino acid comparison-based approaches on ancient metagenomic datasets [45]. Despite these efforts, benchmarking needs revisiting for the following reasons. First, Velsko et al., (2018) [44] and Eisenhofer et al., (2019) [45] primarily focused on the damage patterns arising from deamination and fragmentation in ancient DNA but not from modern DNA contamination. Second, a significant proportion of reads are rendered “unclassified” due to the inability of most of the profilers to classify damaged metagenomic reads, whose preponderance increases in ancient metagenomes with the passage of time. For assessment, a composite accuracy metric, such as F1-score needs to be used, which is the harmonic mean of precision and sensitivity. It not only accounts for misclassifications but also for unclassified reads (as false negatives in the computation of sensitivity). This provides a holistic evaluation of each profiler’s performance. Third, the level of ancient DNA damage can vary due to multiple factors, such as age, location, and sample handling. In several studies, the performance of metagenomic classifiers as a function of the level of DNA degradation in ancient metagenomes was not assessed [42,43,44,45]. Furthermore, as several new metagenomic classifiers have been developed in recent years, a benchmark update with new methods is needed and a comprehensive assessment needs to be performed as suggested above.

To address this knowledge gap and advance the field of ancient metagenomics, in particular, we performed a comprehensive assessment of currently used metagenomic profilers at different levels of DNA damage patterns in simulated ancient dental calculus metagenomes. Our analysis delves even deeper by simulating damage patterns arising due to not only deamination and fragmentation but also modern DNA contamination (both human and microbial contaminations) This study includes a variety of new and updated algorithms that have been frequently used in the taxonomic profiling of modern metagenomic data.

## 2. Materials and Methods

### 2.1. Simulation of Ancient Metagenomic Data

Metagenomes with and without damages were generated using Gargammel [46] and were retrieved as FASTQ files. The complete data represented four sets of simulations (Supplementary File S1). Each of these sets comprised 5 samples each for simulated data with high damage, medium damage, low damage, and no damage (modern metagenome). In total, there were 80 simulated samples, 20 samples in each set. Set 1 contained samples with varying levels of deamination representing different levels of damage, from none to high damage, and with fragments of length with a log-normal distribution same across all samples (parameters set to—loc 4.106—scale 0.359 in Gargammel) and with no human contamination (modern) and 1% ancient human contamination in each of the high damage, medium damage, and low damage datasets. This was simulated by changing the damage argument which includes four parameters: nick frequency, length of overhanging ends, probability of deamination in double-stranded parts, and probability of deamination in single-stranded parts. All these parameters were changed from high damage to no damage (modern datasets) as allowed by the standard aDNA damage models of Gargammel (Table 1). Set 2 contained samples with varying levels of deamination and fragmentation but without contamination with modern human DNA. This was performed by changing the -rl flag from 40–125 bp simulating high damage to modern datasets, respectively (Table 1). Set 3 samples were generated by adding modern human contamination to the samples of Set 2 (Table 1). This was performed using a comp flag and by successively decreasing human contamination from 80% (high damage datasets) to 0% (modern datasets). Set 4 was generated by using the same flag and successively decreasing the microbial contamination from 80% in high damage datasets to no contamination in modern datasets (Table 1). Note that 80% contamination is very rare per the prevailing knowledge of the ancient metagenomics datasets, however, as is typical with benchmarking studies, we assessed here the performance of profilers as a function of contamination that included the extremities in addition to the realistic estimates of the contamination. A total of five soil microbial species were used in Set 4 for microbial contamination (details are provided in Supplementary File S1). The simulated reads originated from 100 different microbial genomes each contributing equivalently, which were selected based on bacterial communities known to be dwelling in dental calculus according to the Human Oral Microbial Database (www.homd.org (accessed on 26 September 2023)). For the benchmarking purpose, the assumption of equal abundance of the species could be a simplification, which, however, provides a clear reference point against which the classifiers’ abilities to accurately classify species can be measured. Additionally, for the benchmarking purpose that strives to be not affected by peculiarities of a specific metagenome, this attempts to remove any bias that might be introduced if certain species were more abundant than others, potentially skewing the results in favor of classifiers better suited to identifying the prevalent species. Illumina HiSeq2500 was used as the sequencing platform for this analysis. The commands used for the simulation are provided in Supplementary File S1.

### 2.2. Taxonomic Profiling

The metagenome profilers were assessed on simulated metagenomic samples and were grouped based on their algorithm for taxonomic assignment (Table 2). DNA-to-DNA comparison methods include Kraken2 (version 2.1.3) and KrakenUniq (version 1.0.2), which match metagenomic reads to the DNA sequences in the microbial database. For the Kraken2 usage, a custom database of bacterial and archaeal genomes was constructed. FASTA files were provided as input to the Kraken2 and relative abundance report files were generated. Genus and species-level relative abundance data were extracted from the report file through shell scripting. Classified and unclassified reads were obtained in separate files.

Similar to Kraken2, for KrakenUniq, a custom database of bacterial and archaeal genomes was generated. FASTA files of the samples were inputted to KrakenUniq and the genus and species level relative abundance data were extracted from the report file through shell scripting. Bracken (version 2.5.0) used the Kraken2 database and used the reports generated by Kraken2 for its analysis.

Kaiju (version 1.8.2) represents the DNA-to-protein profiler, which compares each of the six frame translations of metagenomic reads to protein sequences in the microbial database. The database of Kaiju was downloaded from https://kaiju.binf.ku.dk/server (accessed on 26 February 2021), and we further downloaded the RefSeq database (dated 26 February 2021).

mOTUs (version 2.6.1) and Metaphlan4 (version 4.0.4) represent marker-based profilers, which align metagenomic reads to marker sequences unique to different microbial taxa in the database. We generated the standard database for both of these tools. An additional flag of –read_min_length was used to specify the minimum read length.

All commands for data simulation and processing and running the profilers are provided in the GitHub repository at https://github.com/VaidehiPusadkar/Benchmarking-profilers-on-ancient-metagenome (accessed on 27 September 2023).

### 2.3. Performance Evaluation

Sensitivity and precision were calculated as described in other benchmarking studies [22,47]. In some cases, a genome may not have taxonomic labels for all ranks (species, genus, family, etc.). Sensitivity and precision were calculated for species and genus-level taxonomic ranks. Sensitivity (e.g., genus-level) was computed as A/B where A is the number of reads with the genera correctly assigned by a method and B is the total number of reads of known genera. Precision (e.g., genus-level) was calculated as X/(X + Z), where X is the number of reads with genera correctly assigned by a method, and Z is the number of reads with an incorrect genus assignment by the method. F1 score is the commonly used metric for evaluating the performance of classification models, especially when there are imbalanced datasets. Precision here is the proportion of all classifications at a taxonomic level (species or genus) made by a method that are correct. Sensitivity here is the proportion of all reads in the test set that were correctly classified at the desired taxonomic level (species or genus) by a method. The best performing profilers strive to balance precision and sensitivity to attain a high F1 score. The F1 score was calculated as:

F1 score = 2 × (Precision × Sensitivity)/(Precision + Sensitivity).

## 3. Results

We benchmarked six metagenomic profilers; a comparative assessment of the profilers was performed based on the accuracy metrics precision, sensitivity, and F1 score (see Section 2). DNA-to-DNA profilers included Kraken2 (and its add-on version for more accurate abundance estimation, namely, Bracken) and KrakenUniq [22,23,48] DNA to protein profiler included Kaiju [28], and DNA to markers profilers included MetaPhlAn4 [49] and mOTUs [32] (Table 2). The latest versions of the aforementioned profilers were used.

### 3.1. Effects of Deamination on Metagenomic Read Classification

Ancient metagenomic DNA is susceptible to deamination [13,50,51], resulting in guanine to adenine and cytosine to thymine substitutions. These substitutions are localized near the end of the fragments with a substantial decrease in the substitutions further along the DNA sequenced. Although portions of sequences that are deaminated will make classification difficult, however, the phylogenetic signals exploited by the profiling methods may still be discernible in a large fraction of sequences since the effects of the deamination diminish away from the ends. To evaluate this, we simulated deamination generating four metagenomic datasets by successively decreasing the rate of deamination (from 80% to no deamination) with five replicates in each dataset of high damage (80% deamination), medium damage (40% deamination), low damage (10% deamination), and no damage (0% deamination) (Table 1). All other properties (such as fragment length and contamination) were kept the same across all four samples (see Section 2). We assessed the performance of the profilers by comparing the relative abundance of the taxa outputted by each profiler (at the genus and species levels) with the ground truth. The performance evaluation metrics used were precision, recall, and F1 score, and the results for each dataset were averaged over the five replicates. The precision of DNA-to-DNA based profilers (Kraken2 and KrakenUniq) was higher compared to that of the profilers of other types, even for highly damaged reads at both genus and species levels (indicating their lower false classifications), except Bracken that tends to produce more false classifications (Supplementary Figure S1). However, the sensitivity (recall) of these profilers was lower compared to that of the DNA-to-markers based profilers (MetaPhlAn4 and mOTUs) (Supplementary Figure S1). The lower sensitivity of Kraken2 and KrakenUniq is due to large numbers of reads that remain unclassified for all datasets used. Notably, post-processing of Kraken2′s output by Bracken improved its sensitivity at the genus and species levels (Supplementary Figure S1).

**Figure 1:**
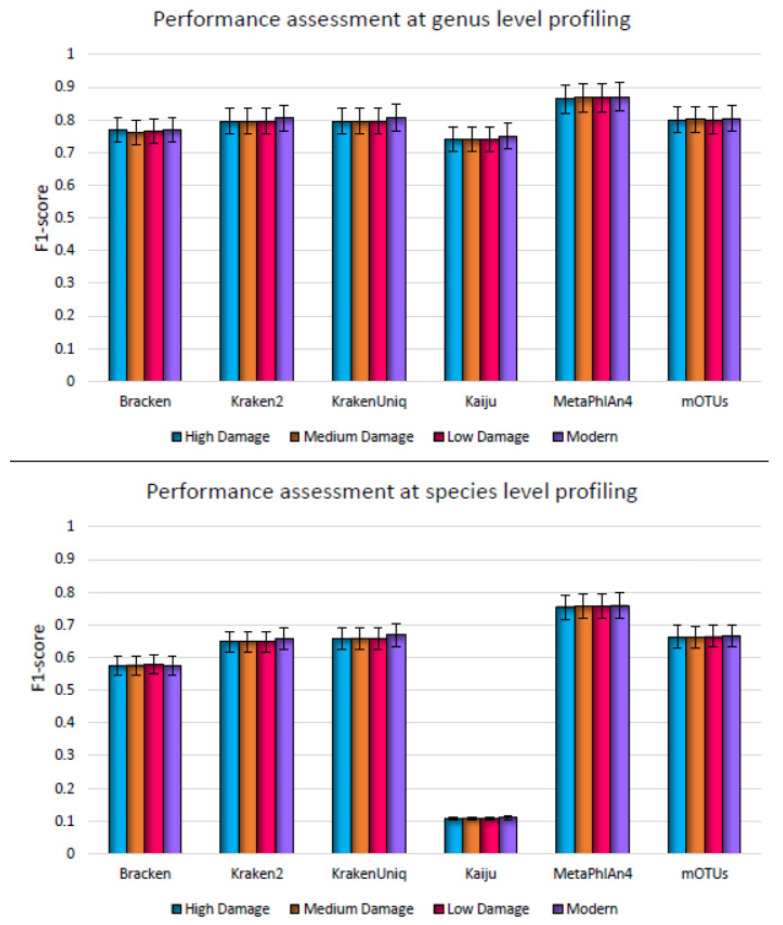
Genus level (**top**) and species level (**bottom**) F1 score for metagenome profilers assessed on synthetic datasets simulating high damage, medium damage, low damage, and no damage, respectively. Here damage refers to deamination and F1 score is the average over five simulated replicates.

The overall accuracy of the profilers was assessed using the F1 score, which is the harmonic mean of precision and sensitivity. The overall performance scores of all the profilers did not change significantly as the deamination was increased, simulating modern to high-damage samples at the genus and species levels (Figure 1). The overall performance at the species level was lower than that at the genus level as expected, though the patterns (performance variation when assessed on datasets with high to no damage) were similar. Interestingly, the species-level performance of MetaPhlAn4 (F1 score ~0.86) was not noticeably lower than the genus-level performance across the damage levels as compared to the other profilers. mOTUs and DNA-to-DNA profiling methods, such as Kraken2 and KrakenUniq, had the second highest F1-score (~0.80) on high damage datasets, which did not decrease with an increase in the deamination. This shows their capability to accurately classify taxa in these high damage datasets as well. DNA-to-protein profiler Kaiju had the lowest F1 score amongst all the profilers.

### 3.2. Effects of the Combination of Deamination and Sequence Fragmentation on Metagenomic Read Classification

**Figure 2:**
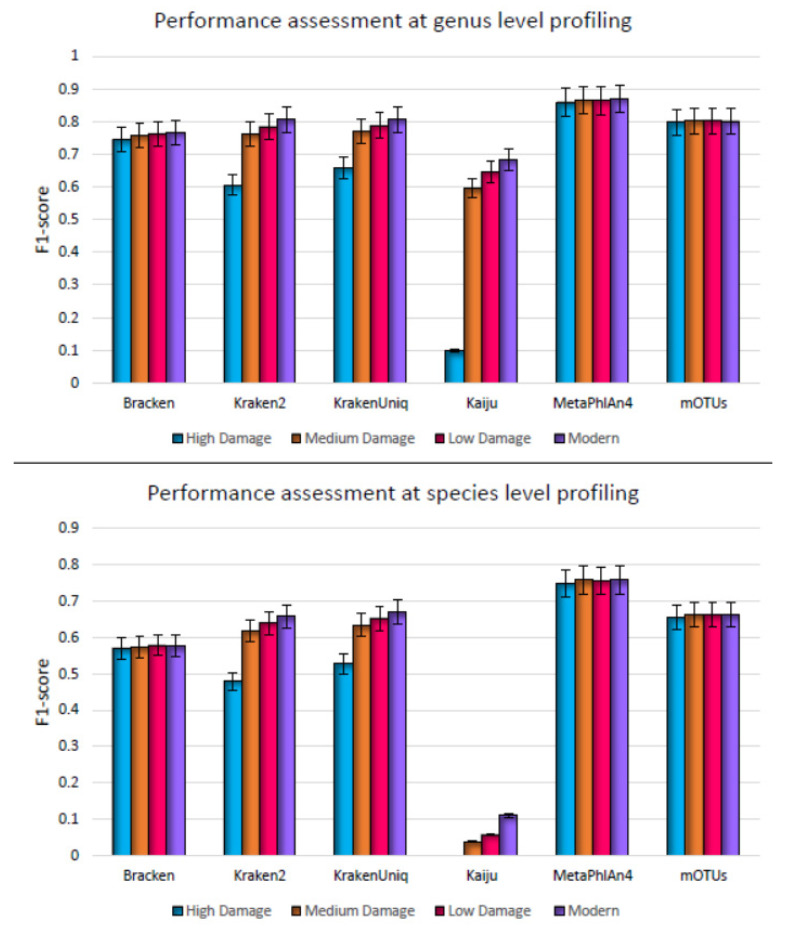
Genus level (**top**) and species level (**bottom**) F1 score for metagenome profilers assessed on synthetic datasets simulating high damage, medium damage, low damage, and no damage, respectively. Here damage refers to deamination and fragmentation and F1 score is the average over five simulated replicates.

A significant challenge in ancient metagenome characterization is the prevalence of short DNA fragments in ancient samples, resulting in shorter reads sequenced by current sequencing technologies. DNA fragmentation in ancient samples happens due to DNA hydrolysis and cross-linking due to condensation and oxidation of pyrimidines that do not allow extension of DNA during polymerase chain reaction (PCR) [52]. As a consequence of this, the metagenomic profilers are challenged with insufficient conservation signals encoded in sequences, which may result in the erroneous taxon classification. Note that most high throughput sequencing technologies generate shorter reads regardless of sample age [20]. Modern metagenome profilers have, therefore, been designed for the analysis of short reads in a reasonable time. However, several of these profilers have not yet been tested on ancient metagenomic samples comprehensively. Therefore, to evaluate the impact of fragmentation on the read classification, fragment length for simulated deaminated samples was varied from 40 bp (high damage datasets) to 125 bp (modern datasets). The fragment lengths were chosen based on the respective averages from recent large-scale ancient and modern sequencing studies [13,53,54] (Table 1). In contrast to deamination alone, we now observed a substantial decline in the performance of the profilers on high damage datasets at both genus and species levels (Figure 2, Supplementary Figure S2). Specifically, the decline in the performance of the DNA–protein profiler, Kaiju, on high damage datasets was more pronounced (F1 score declined to 0.09 from 0.68 on no damage datasets, a 59% decrease) compared to the other profilers. As the read length decreased, the number of reads rendered unclassified by Kaiju increased sharply, decreasing substantially its sensitivity at both the genus and species levels (0.05 and 0.02, respectively) and thus resulting in lower F1 scores. Application of DNA–DNA tools (Kraken2, KrakenUniq, and Bracken) to simulated datasets also showed a decreasing trend in F1 score with damage, however, the decline in F1 score was not so pronounced as Kaiju. Specifically, for Kraken2, the F1 score for high to no damage datasets ranged from 0.61 to 0.80, for KrakenUniq, it ranged from 0.65 to 0.8076, and for Bracken, it ranged from 0.74 to 0.76, at the species level. This was more prominently manifested with high damage datasets. The precision of Kraken2 and KrakenUniq still remained the highest among all the profilers at all damage levels (>0.95). However, their sensitivity decreased as higher percentage of reads, particularly of shorter fragment lengths, were left unclassified. Bracken, although with relatively low overall performance, was least sensitive to the damage. On the other hand, DNA-to-marker based profilers (mOTUs and MetaPhlAn4) were not impacted by the damage, at any level. F1 score of MetaPhlAn4 ranged from 0.86 to 0.87 and for mOTUs was 0.80 at all the levels of DNA damage. These profilers use clade-specific marker genes that are typically shorter in length [32,49]. These are specifically designed to align short metagenomic sequences to marker gene sequences. Further, their algorithms allow a certain level of mismatches in the reads and the taxon call is based on read coverage of a marker rather than simply a read match to a reference sequence. These profilers can, therefore, classify short reads even when they are fragmented. Furthermore, these methods balanced well the precision and sensitivity tradeoff. As these profilers did not yield output for high damage datasets at their default setting, we had to incorporate additional flags specifying minimum read length (40 bp for high-damage data).

### 3.3. Effects of the Combination of Deamination, Sequence Fragmentation, and Human Contamination on Metagenomic Read Classification

**Figure 3:**
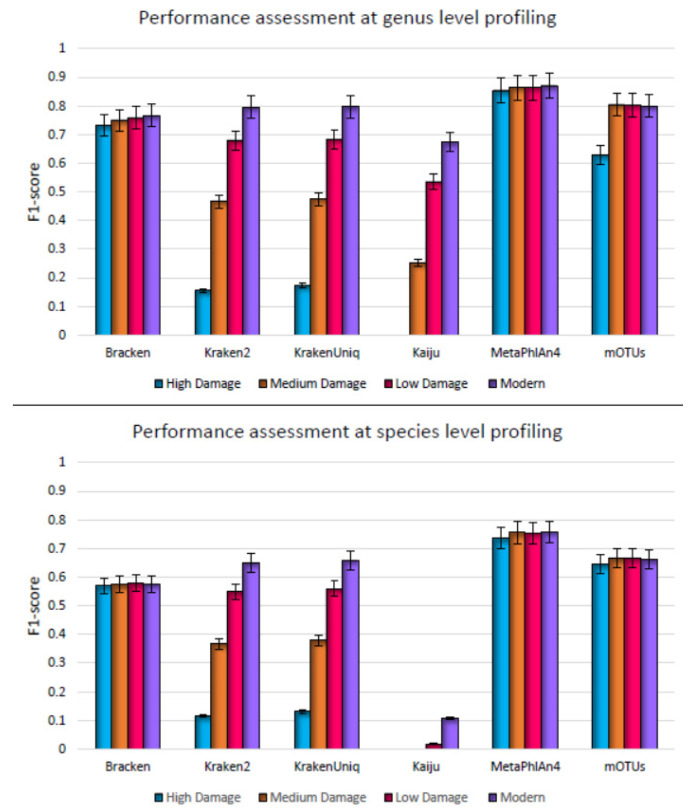
Genus level (**top**) and species level (**bottom**) F1 score for metagenome profilers assessed on synthetic datasets simulating high damage, medium damage, low damage, and no damage, respectively. Here damage refers to deamination, fragmentation, and modern human contamination and F1 score is the average over five simulated replicates.

One of the challenges encountered in ancient DNA analysis is the contamination of ancient microbial samples with modern human DNA. The reads that align to a specific target genome sequence with an adequate level of similarity are called endogenous reads (that is, originating from the target species). On the other hand, exogenous DNA in the ancient microbial samples could be from soil burial matrix, lab reagents, handling of the DNA from archeological sources, and lab equipment [18,55] with traces of DNA from modern humans [55,56,57,58]. These contaminant sequences are randomly added to the samples and differ widely among samples. It is, therefore, important to understand how contamination of ancient metagenomic samples with modern DNA would affect the classification of microbial species by different profilers. To this end, we challenged the metagenome profilers by simulating contamination on top of deamination and fragmentation in the test metagenomic datasets. Human (originating from genome build GRCh38.p13) contamination of varying proportions was added to each sample type (Table 1). Not unexpectedly, we observed a more pronounced decline in the performance of the profilers as the damage increased (Figure 3). The F1-score of Kraken2, KrakenUniq, and Kaiju decreased sharply as the damage level was augmented (from modern to high damage datasets, Figure 3). Their sensitivity also decreased with increasing contamination and decreasing fragment size (Supplementary Figure S3). This drop in the overall performance accuracy (F1 score) indicates that as the contamination is incorporated in the aDNA samples, the profilers are not able to classify the reads at the desired taxonomic resolution. Bracken, despite reporting more classified reads than by the other DNA-to-DNA comparison methods at high damage, does not directly perform read classification. Rather, it uses a probabilistic re-distribution approach to estimate the true abundance profile for a metagenomic sample based on initial read classification generated by Kraken. This allows Bracken to assign the unclassified reads to different taxa based on their estimated abundance. As a result of its probabilistic approach, Bracken assigns a read to a species that yields the highest probability for the read to have originated from it rather than the exact match (as in Kraken), due to which the reads that are left unclassified by Kraken are classified by Bracken [20]. Nevertheless, Kraken2 and KrakenUniq still maintained their precision by incurring low misclassifications (above 0.9 at high damage). On the other hand, the precision and sensitivity of Metaphlan4 and mOTUs were well balanced for all the datasets (modern to medium damage) and the contamination did not affect the performance of DNA-to-markers based profilers. Consequently, their overall F1 scores remain unaffected by all three damage patterns. Metaphlan4 outperformed the other methods, attaining the highest F1 score on high damage data sets (0.85 at genus level and 0.73 at species level). Note that instead of relying on exact matches (as with DNA-to-DNA based profilers), DNA-to-markers methods use a profiling approach based on the similarity of a read to the clade specific marker genes. Similarity inference is based on a nucleotide identity threshold that allows changes in the read sequence to a certain extent, and furthermore, the taxonomic identity is based on the overall coverage of a marker by the reads mapping onto it. These factors may render these methods less sensitive to the aforementioned damage patterns.

### 3.4. Effects of the Combination of Deamination, Sequence Fragmentation, and Microbial Contamination on Metagenomic Read Classification

In addition to the potential for human contamination, the integrity of ancient metagenomes can also be compromised by environmental microbes present in the burial matrix or excavation site, or any other locations from which the archaeological samples are collected. This contamination can occur via contact with tools, clothing, or laboratory equipment that may carry contemporary DNA from soil or other sources [59]. This can affect the taxonomic profiling potentially leading to misclassification of endogenous microbes or misrepresentation of ancient microbiome, and hence affecting the interpretation of ancient metagenome in different samples [20,60]. To assess the impact of microbial contamination on the performance of multiple profilers, we simulated metagenome datasets, each characterized by a distinct level of microbial damage. For contamination, we chose five species that are prevalent in soil: *Streptomyces griseus*, *Pseudomonas putida*, *Bacillus subtilis*, *Agrobacterium rhizogenes*, and *Agromyces aureus*. Following a similar approach to simulation of human contamination, we increased the contamination-related damage level from 0 (modern datasets) to 80% (high damage datasets).

**Figure 4:**
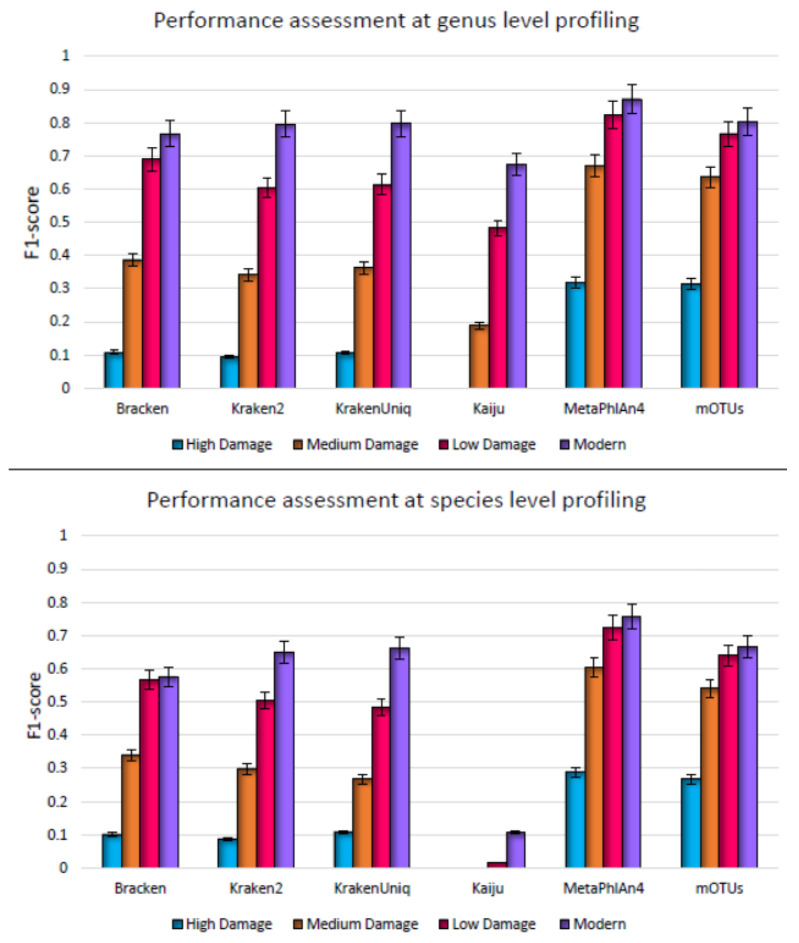
Genus level (**top**) and species level (**bottom**) F1 score for metagenome profilers assessed on synthetic datasets simulating high damage, medium damage, low damage, and no damage, respectively. Here damage refers to deamination, fragmentation, and microbial contamination and F1 score is the average over five simulated replicates.

Notably, it was found that microbial contamination-based damage had the most pronounced impact on the performance of each profiler. The F1-score of each profiler exhibited a significant decrease with increasing level of contamination (Figure 4). Even Kraken2 and KrakenUniq, which displayed high precision with the other damage patterns, declined substantially in precision when challenged by microbial contamination. This can be attributed to the selection of the database used in our analysis. In the prior analysis, when a custom database that included bacterial and archaeal species was used, Kraken2 and KrakenUniq were effective in robust profiling of metagenomes with human contamination. However, this was not so in the context of microbial contamination and with this database. Consequently, this led to a remarkable decrease in the overall F1 score as compared to the previous simulation experiment. Further, the trend observed in the performance of Bracken, mOTUs, and MetaPhlAn4 that maintained consistent F1-score across the range of human contamination level was not replicated for microbial contamination. Compared to the performance on metagenomes with human contamination (Figure 3), the species level overall accuracy (F1 score) decreased from 0.73 to 0.28 for MetaPhlAn4, from 0.64 to 0.27 for mOTUs, and from 0.56 to 0.1 for Bracken when microbial contamination was considered (high damage level, Figure 4). The precision and sensitivity of all profilers steadily decreased with increasing microbial contamination (Supplementary Figure S4). MetaPhlAn4 outperformed the other profilers in classifying the reads, with highest F1 score across all damage levels (Figure 4). Kaiju had the highest number of unclassified reads. It failed to classify reads at the high damage level, and even at the medium damage level (50% contamination) when species profiling was assessed. In light of these findings, it becomes evident that recognizing and effectively addressing contamination, particularly, the microbial contamination, need to be considered a critical initial step in all analyses of ancient microbiomes. Proper decontamination procedures are imperative to ensure fidelity of inferences and insights gained from ancient metagenomic data analysis.

## 4. Discussion

This study advances over the previous benchmarking studies by assessing the effects of not just deamination and fragmentation but also modern human and environmental contaminations on the performance of cutting-edge profilers. Furthermore, a multi-layered approach is taken that provides insights into each profiler’s performance at three different levels of damage. We recognized that the issue of unclassified reads is important in ancient metagenome studies due to different damage patterns observed in ancient samples that render a large number of reads labeled unclassified by profilers. This was accounted for in our assessment through the F1-score metric.

Deamination (cytosine to thymine and guanine to adenine substitutions at the 5′ and 3′ ends of the sequenced DNA molecules, respectively) had a minimal effect on the performance of the profilers. Challenged with fragmentation and deamination together, the sensitivity of DNA-to-DNA comparison methods (Kraken2 and KrakenUniq) and DNA-to-protein method (Kaiju) decreased significantly with damage (Supplementary Figure S2, Supplementary File S1). Further, after adding human and microbial contaminations to these damaged datasets, we observed a further decline in the sensitivity and F1-score of these tools.

Among the DNA-to-DNA comparison methods, Kraken2 has emerged as a fast and robust metagenomic profiler. Kraken2 searches *k*-mers from metagenomic reads in the database and the lowest common ancestor (LCA) of all the genomes that contain the *k*-mer in the query sequence is identified [22]. The default size of *k*-mer for Kraken2 is 35 nt; each distinct *k*-mer from a read is subjected to the LCA analysis, which renders Kraken2 the ability to classify the reads with higher accuracy than many other profilers despite the deamination and fragmentation of the reads. Deamination has the potential to obfuscate the evolutionary signals encoded within *k*-mers, however, as a large fraction of a read remains insulated from deamination that degrades mainly the regions at the ends of the read, programs such as Kraken can extract the conserved signals in *k*-mers from un-deaminated regions and classify robustly. If deamination could happen across the entire span of the reads, the accuracy can drop precipitously. This was indeed observed when the metagenomic reads were deaminated randomly across their entire span; the *k*-mers extracted from these reads could not get matched to the genomes of their originating species, which resulted in a high proportion of unclassified reads, thus affecting the sensitivity of theses profilers (Supplementary Figure S3). As Kraken uses exact matching of each *k*-mer in the read to the *k*-mers in the reference database, this tends to affect the profiler’s performance when contamination is added to the simulated metagenomes. Further, as expected, the species level performance declines sharply compared to the genus or higher taxonomic level performance. Nevertheless, Bracken, the add-on tool for Kraken2, overcomes the above bottlenecks of Kraken2. It takes as input the Kraken2’s classified and unclassified reads and re-estimates their abundance at desired taxonomic resolutions. However, it also tends to increase false positives, particularly when the read-originating genomes are not represented in the database. If the read-originating genomes are not available in the Kraken2 database, Kraken2 tends to label those as unclassified, however, Bracken assigns those to the genomes that yield the highest similarity score based on its probabilistic approach to abundance estimation. This can increase false positives, particularly at the lower taxonomic levels. Another profiler KrakenUniq, an extension of Kraken, works on exact *k*-mer matching based on the HyperLogLog algorithm. KrakenUniq performed comparably to Kraken2 at the genus level but not so at the species level, establishing Kraken2 as a better profiler for ancient metagenome profiling. Furthermore, the database requirements for Kraken2 are less than those of KrakenUniq [48].

The DNA-to-protein profiler Kaiju translates each of the metagenomic reads to six possible protein sequences (corresponding to six reading frames) and finds their maximum exact matches (MEMs) in the database of annotated proteins from reference genomes. The database used by these profilers contains only microbial proteins. As a result, despite having a low proportion of misclassified reads, they tend to have a high number of unclassified reads. Consequently, their sensitivity is low, particularly when the reads are short and contaminated (Supplementary Figures S2 and S3). Furthermore, the overall performance of Kaiju was lower compared to other tools; our analysis thus indicates that Kaiju is less efficient for ancient metagenome analysis.

Among the DNA-to-marker alignment profilers, mOTUs perform marker gene assignment at the operational taxonomic units (OTUs) level with more than 7500 microbial species in its database. As with other tools, the F1-score of mOTUs was not affected noticeably by increasing levels of DNA damage; however, the overall performance of mOTUs was lower than that of the other profilers, and further, its performance declined from genus to species level profiling even more than that of the other DNA-to-marker based profiler, MetaPhlAn4.

MetaPhlAn4 performs the alignment of metagenomic reads to clade-specific marker genes that have been selected for uniquely assigning the reads to the originating taxa, from species to higher taxonomic levels. The latest version of MetaPhlAn4 spans 236,600 reference genomes and 771,500 metagenomic assembled genomes (MAGs) [49]. The inclusion of MAGs allows classification of reads originating from yet uncharacterized microbial genomes (or draft genomes), that is, from those that are not part of the reference genome database [61]. In fact, multiple studies on ancient metagenomes have used MAGs that are obtained from these studies [62]. A recent study employing MetaPhlAn4 reclassified ancient metagenomic samples previously classified by MetaPhlAn3 and revealed an increase in observable microbial diversity by quantifying uncharacterized species [49]. This brings a light to novel directions that can address the problem of unclassifiable reads due to database limitations (for both modern and ancient metagenomes). Further, in addition to not being sensitive to read length, MetaPhlAn4 also remained largely unaffected by the contamination of the sequences (Figure 2 and Figure 3). This is because rather than relying on exact matches for the metagenomic read *k*-mers, MetaPhlAn4 finds the best match of the reads with the cataloged marker genes (based on a local sequence alignment, with identity threshold set at 75% allowing for mismatches, which allows toleration of contamination to an extent). This may, however, come with the risk of a decrease in precision because of misclassifications, which is not the case with *k*-mer based profilers. MetaPhlAn4 uses nucleotide BLAST (blastn) with a default E-value threshold of 1 × 10^−6^ to align reads to marker genes. When multiple matches occur between a read and markers from different clades, only the top hit is taken into consideration [31]. Further, taxonomic inference is based on read coverage of the marker genes. Such an approach allows marker-based methods to handle variations, mutations, or sequencing errors, and even contaminations in the reads while still providing robust taxon abundance estimations. Consequently, the performance of DNA-to-markers based methods remains unaffected with an increase in the damage of metagenome. When the microbial reads were subjected to human contamination, markers-based methods were not able to identify human reads as these reads may not span or contain human markers sequences. Their database requires low memory storage (2–3 GB) and is easy to generate. Note that, this profiler, by its design, is an abundance estimation program; it does not perform taxonomic classification of each read in a metagenomic sample, and therefore, it cannot be used to bin reads of potentially the same origins or assemble genomes based on binning or clustering. Further, it does not provide an option for a custom database, which is provided by multiple other profilers (Table 2). Nevertheless, several positive aspects of this profiler make it an efficient tool for ancient metagenomic studies.

Contamination of ancient metagenomes presents a significant challenge to analysis and interpretation of ancient microbial samples. From the collection of archeological specimens to the sequencing of their genomes, there are multiple stages in the process that can inadvertently contribute to the contamination of the endogenous ancient DNA. Such contaminants, originating from diverse sources, such as the soil burial matrix and the human skin of those involved in sample handling, have an uncanny ability to persist despite rigorous cleaning and sterilization procedures, thereby exerting a lasting influence on metagenomic profiles derived from sequencing efforts. Our study highlights the impact of these residual microbes on the metagenomic profiles generated by different profiling methods. Therefore, it is critical to differentiate between contaminant microbial signatures and the authentic signatures originating from an environment or host to ensure the precise reconstruction of host-associated microbial profiles. There are some preprocessing (standards and preventive measures) pipelines designed to handle ancient samples with efficiency and to improve their quality [18,60]. However, the metagenome profilers used in these pipelines that assign a taxonomic identity to each read need to be improved for differentiating exogenous DNA from endogenous DNA. For modern human contamination, multiple efforts are made to quantify and discard most of these contaminants [58,63]. However, these efforts do not guarantee complete removal of these contaminants. Our study has established that DNA-to-markers based methods are efficient in classifying metagenomic reads even with existing human contamination. Microbial contamination poses a challenge to all profilers to correctly identify the endogenous species from the mixture with other microbial species. This could be even more challenging if the exogenous microbes are also ancient. These can be reduced by excavating and storing new specimens using ad hoc precautions to prevent contamination or growth of microbial contaminants on the samples.

The contamination-induced damage was found to exert the most significant impact on the ability of the profilers to classify reads from ancient metagenomes. In light of these findings, we present a set of guidelines aimed at improving the classification accuracy of profilers when dealing with damaged metagenomes. Profilers with custom database-building options may be useful in only targeting endogenous reads isolated from a particular source. This may affect the sensitivity but can improve the precision by reducing false classifications. Building reference databases that account for ancient DNA damage patterns can significantly improve classification accuracy. DNA-to-DNA and DNA-to-marker based methods were shown to have complementary strengths in terms of precision and sensitivity. They could thus be used in combination; future studies could focus on combination or ensemble approaches that could further elevate the bar in the taxonomic classification of aDNA. Incorporating steps in the read processing pipeline that can effectively target reads with damage-related signatures may render an improved profiling of ancient metagenomes. Further, by incorporating algorithms that can detect contaminations and their sources, the profilers can diminish the sources of misclassifications and thus augment their performance. In fact, bioinformatics tools that allow for detection and quantification of contaminations through post hoc comparisons following metagenome sequencing already exist and could be readily incorporated within a profiling pipeline. These methods rely on strict filtering based on statistical methods. For example, tools such as Source Tracker [64] and Meta-SourceTracker [65] were developed to track the source of metagenome reads in 16S rRNA and whole genome shotgun sequencing data, respectively. Users are recommended to use those tools to estimate the percentage of reads contaminated. Collaborations between archaeologists and aDNA researchers could go a long way in crafting efficient protocols designed to diminish and preempt DNA contamination systematically.

## 5. Conclusions

In this study, we considered three main damage patterns, namely, deamination, fragmentation, and contamination, which have been observed in the ancient metagenomic data to first simulate ancient metagenomes with such patterns and then evaluate several metagenomic profilers on the simulated metagenomes. Our results provide insights into the strengths and weaknesses of different profilers, specifically as the performance is assessed as a function of DNA damage. Kraken2 (used with Bracken for species-level abundance estimation) and MetaPhlAn4 emerged as the most effective tools for metagenome profiling. Ancient metagenome studies span several disciplines, including epidemiology, microbiology, and archaeology, and can provide exciting perspectives on modern metagenomes, for example, on the evolution of novel traits in microbial communities. Therefore, it becomes imperative for metagenomic profilers to be effective in profiling both modern and ancient metagenomic reads with reduced computation and reasonable runtime. Future studies could focus on developing even more efficient tools that can exploit MAGs in the classification of ancient metagenomic reads in order to uncover yet unknown species in ancient microbiomes.

## Figures and Tables

**Table 1:** Simulated metagenomic datasets and their properties.

Data Sets	Ancient Species Contamination (%)	Number of Metagenomic Reads	Deamination (%)	Fragment Length (bp)	Modern Species Contamination (%)
High Damage	1	5 million	**80**	**40**	**80**
Medium Damage	1	5 million	**40**	**70**	**50**
Low Damage	1	5 million	**10**	**100**	**20**
Modern	0	5 million	**0**	**125**	**00**
			Simulation 1		
			Simulation 2		
			Simulation 3		
			Simulation 4		

Different simulation sets are distinguished by color coding– Simulation set 1 in gold (damage by deamination), Simulated set 2 in green (damage by deamination and fragmentation), and Simulation sets 3 and 4 in brown (damage by deamination, fragmentation, and contamination). Parameters that were changed in the simulations are shown bold-faced.

**Table 2:** List of metagenome profilers that were benchmarked with their details. Memory (RAM) requirements depend on the chosen database. Different types of profiling methods are distinguished by different color backgrounds.

Type of Profilers	Profilers	Custom Database	Memory Requirement (Depends on the Chosen Database)
DNA-to-DNA Profilers	Bracken	Yes (Same as Kraken2)	<1 Gb
	Kraken2	Yes	36 Gb (Custom database)
	KrakenUniq	Yes	200 Gb (Custom database)
DNA-to-Protein Profiler	Kaiju	Yes	25 Gb
DNA-to-Marker Profilers	MetaPhlAn4	No	2 Gb
	mOTUs	No	2 Gb

## Data Availability

Simulation data were produced using scripts made available in the GitHub repository at https://github.com/VaidehiPusadkar/Benchmarking-profilers-on-ancient-metagenome (accessed on 27 September 2023), which includes the codes for data simulation, processing, and running the profilers.

## Supplementary Materials

The following supporting information can be downloaded at: https://www.mdpi.com/article/10.3390/microorganisms11102478/s1, Figure S1: (a) Genus level precision and sensitivity averaged over five simulated data of high damage, medium damage, low damage, and modern data sets each based on deamination, (b) species-level performance precision and sensitivity, averaged over five simulated data of high damage, medium damage, low damage, and modern data sets each based on deamination. Figure S2: (a) Genus level precision and sensitivity, averaged over five simulated data of high damage, medium damage, low damage, and modern data sets each based on deamination + fragment length, (b) species-level performance precision and sensitivity, averaged over five simulated data of high damage, medium damage, low damage, and modern data sets each based on deamination + fragment length. Figure S3: (a) Genus level precision and sensitivity averaged over five simulated data of high damage, medium damage, low damage, and modern data sets each based on deamination + fragment length + modern human contamination, (b) species-level performance precision and sensitivity, averaged over five simulated data of high damage, medium damage, low damage, and modern datasets each based on deamination + fragment length + modern human contamination. Figure S4: (a) Genus level precision and sensitivity averaged over five simulated data of high damage, medium damage, low damage, and modern data sets each based on deamination + fragment length + modern human contamination, (b) species-level performance precision and sensitivity, averaged over five simulated data of high damage, medium damage, low damage, and modern data sets each based on deamination + fragment length + modern human contamination. File S1: File providing details of simulations and performance of the tools.
