# Supplementary material for: Benchmarking Metagenomic Classifiers on Simulated Ancient and Modern Metagenomic Data
Source: Microorganisms. 2023 Oct 2;11(10):2478. doi: 10.3390/microorganisms11102478 (PMC10609333; doi:10.3390/microorganisms11102478)
Supplement: Supplementary file 1 [file microorganisms-11-02478-s001.zip › Supplementary figures.pdf]

SUPPLEMENTARY FIGURES

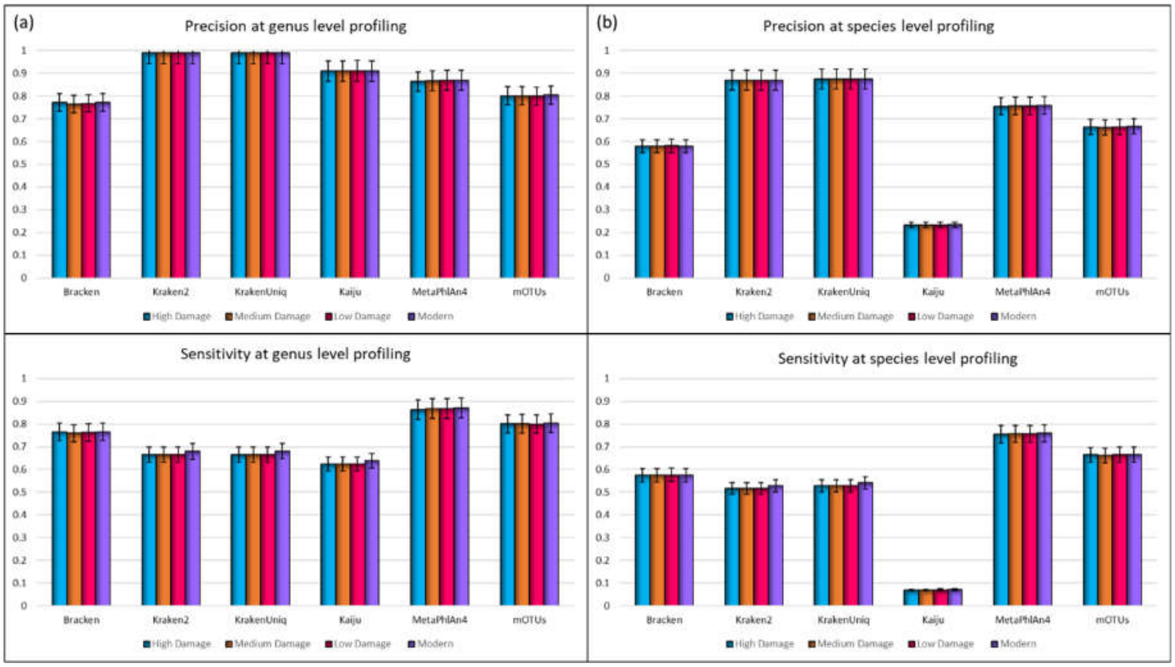

Figure S1: (a) Genus level precision and sensitivity averaged over five simulated data of high damage, medium damage, low damage, and modern data sets each based on deamination, (b) species-level performance precision and sensitivity, averaged over five simulated data of high damage, medium damage, low damage, and modern data sets each based on deamination.

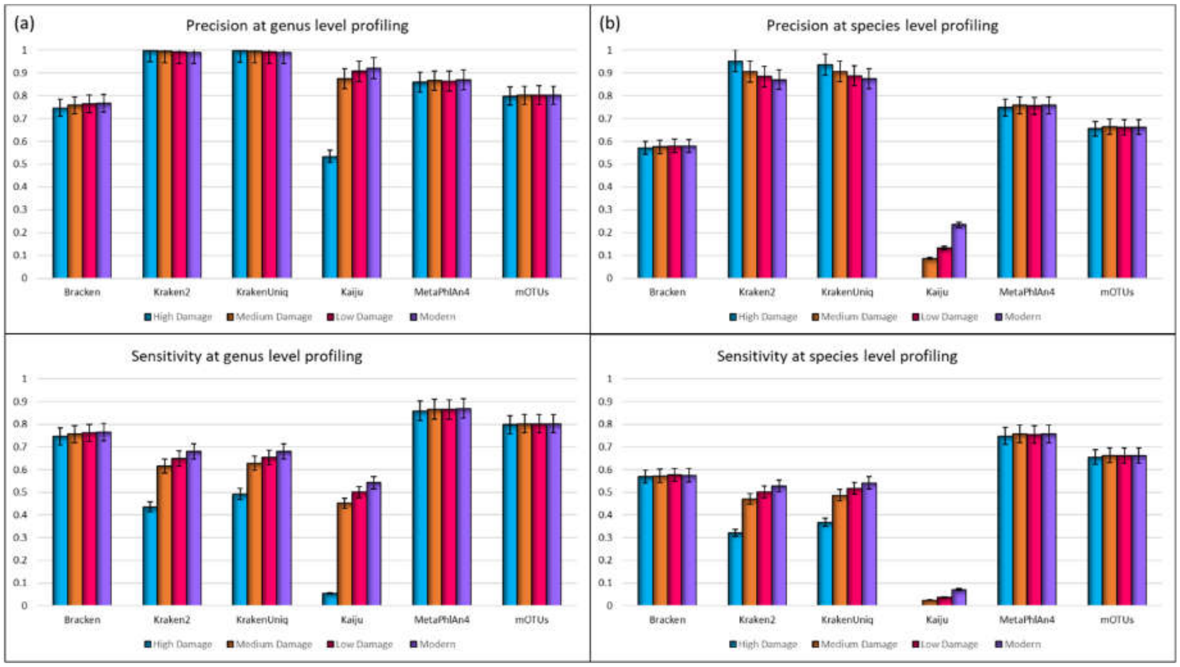

Figure S2: (a) Genus level precision and sensitivity, averaged over five simulated data of high damage, medium damage, low damage, and modern data sets each based on deamination + fragment length, (b) species-level performance precision and sensitivity, averaged over five simulated data of high damage, medium damage, low damage, and modern data sets each based

on deamination + fragment length.

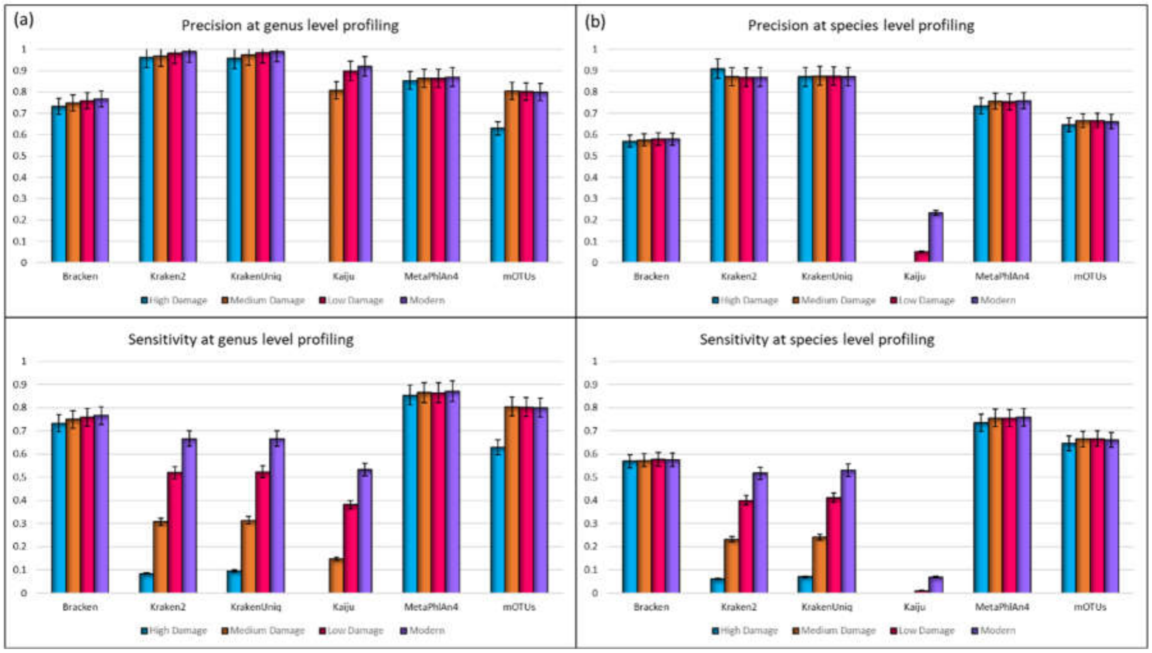

Figure S3: (a) Genus level precision and sensitivity averaged over five simulated data of high damage, medium damage, low damage, and modern data sets each based on deamination + fragment length + modern human contamination, (b) species-level performance precision and sensitivity, averaged over five simulated data of high damage, medium damage, low damage, and modern datasets each based on deamination + fragment length + modern human contamination.

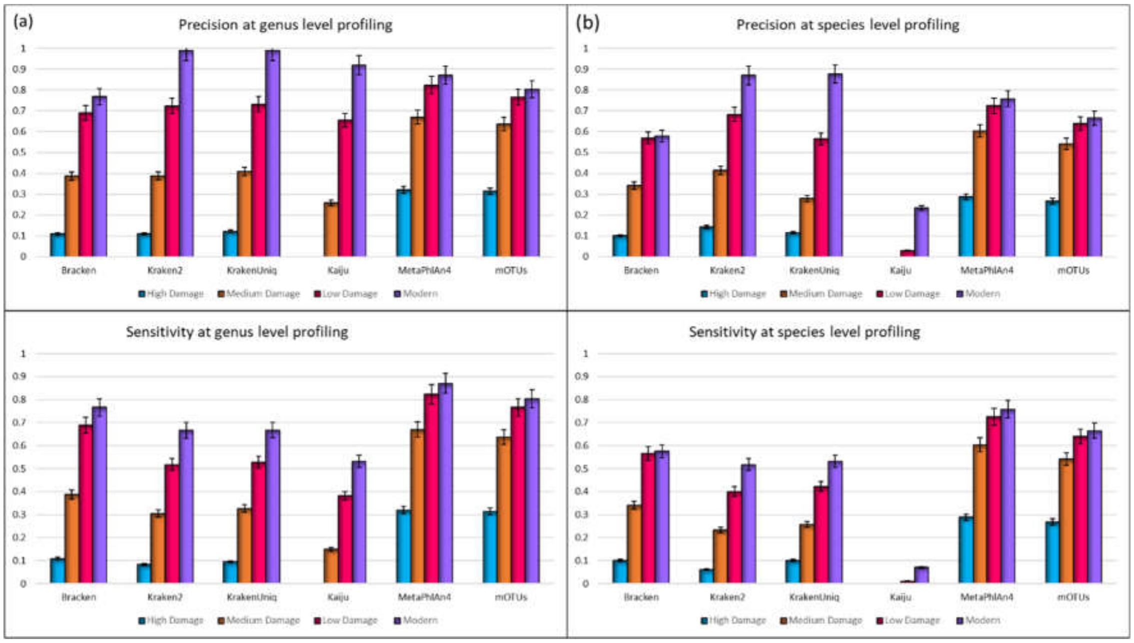

Figure S4: (a) Genus level precision and sensitivity averaged over five simulated data of high damage, medium damage, low damage, and modern data sets each based on deamination + fragment length + modern human contamination, (b) species-level performance precision and sensitivity, averaged over five simulated data of high damage, medium damage, low damage, and

modern data sets each based on deamination + fragment length + modern human contamination.
File S1: File providing details of simulations and performance of the tools.
